# Microglial activation induced by brain trauma is suppressed by post-injury treatment with a PARP inhibitor

**DOI:** 10.1186/1742-2094-9-31

**Published:** 2012-02-15

**Authors:** Joana C d'Avila, Tina I Lam, Deborah Bingham, Jian Shi, Seok Joon Won, Tiina M Kauppinen, Stephen Massa, Jialing Liu, Raymond A Swanson

**Affiliations:** 1 Depts. of Neurology, Veterans Affairs Medical Center and University of California, San Francisco, California 94121, USA; 2 Depts. of Neurosurgery, Veterans Affairs Medical Center and University of California, San Francisco, California 94121, USA; 3 Laboratório de Imunofarmacologia, Instituto Oswaldo Cruz, FIOCRUZ, Av. Brasil 4365, Rio de Janeiro, Brazil 21045-900

**Keywords:** Astrocyte, Behavioral, Forelimb, Inflammation, Microglia, Minocycline, Poly(ADP-ribose) polymerase, traumatic brain injury

## Abstract

**Background:**

Traumatic brain injury (TBI) induces activation of microglia. Activated microglia can in turn increase secondary injury and impair recovery. This innate immune response requires hours to days to become fully manifest, thus providing a clinically relevant window of opportunity for therapeutic intervention. Microglial activation is regulated in part by poly(ADP-ribose) polymerase-1 (PARP-1). Inhibition of PARP-1 activity suppresses NF-kB-dependent gene transcription and thereby blocks several aspects of microglial activation. Here we evaluated the efficacy of a PARP inhibitor, INO-1001, in suppressing microglial activation after cortical impact in the rat.

**Methods:**

Rats were subjected to controlled cortical impact and subsequently treated with 10 mg/kg of INO-1001 (or vehicle alone) beginning 20 - 24 hours after the TBI. Brains were harvested at several time points for histological evaluation of inflammation and neuronal survival, using markers for microglial activation (morphology and CD11b expression), astrocyte activation (GFAP), and neuronal survival (NeuN). Rats were also evaluated at 8 weeks after TBI using measures of forelimb dexterity: the sticky tape test, cylinder test, and vermicelli test.

**Results:**

Peak microglial and astrocyte activation was observed 5 to 7 days after this injury. INO-1001 significantly reduced microglial activation in the peri-lesion cortex and ipsilateral hippocampus. No rebound inflammation was observed in rats that were treated with INO-1001 or vehicle for 12 days followed by 4 days without drug. The reduced inflammation was associated with increased neuronal survival in the peri-lesion cortex and improved performance on tests of forelimb dexterity conducted 8 weeks after TBI.

**Conclusions:**

Treatment with a PARP inhibitor for 12 days after TBI, with the first dose given as long as 20 hours after injury, can reduce inflammation and improve histological and functional outcomes.

## Background

Microglia are the resident macrophages of the CNS [1]. Traumatic brain injury (TBI) leads to tissue disruption and release of molecules from injured and dead cells that elicit microglial and astrocyte activation [2,3]. Activated microglia change morphology, migrate to injury sites, and release reactive oxygen species, nitric oxide, cytokines, metalloproteinases, and other factors with cytotoxic effects. Astrocytes similarly change morphology and acquire a pro-inflammatory phenotype. While this innate immune response can limit the effects of tissue injury and infection, it can also impair recovery and promote secondary neuronal death [1,4-7]. This response requires hours to days to become fully manifest after TBI, a time interval that provides opportunity for therapeutic intervention. Conversely, some aspects of the innate immune response can facilitate later tissue repair and functional recovery [3,8]. Thus, the timing of anti-inflammatory treatment may crucially affect outcomes.

Poly (ADP-ribose) polymerase-1 (PARP-1) is an enzyme involved in both DNA repair and transcriptional regulation [9,10]. PARP-1 is activated by DNA damage and by cytokines such as TNFα. PARP-1 interaction with NF-κB regulates expression of several pro-inflammatory mediators, including proteases, iNOS, ICAM-1, and TNFα [11-14]. PARP-1 activation has been identified in brain after TBI [15-17]. PARP-1 genetic deficiency or enzymatic inhibition suppresses NF-kB- dependent gene transcription in microglia [18] and prevents their morphological transformation, proliferation and migration to injury sites [4,13,19,20]. Several potent PARP inhibitors are now available, and some have been approved for clinical use. Of note, minocycline and related tetracycline derivatives are also highly potent PARP inhibitors [21], and these drugs are also potent suppressers of microglial activation [22-24].

Prior studies have shown that PARP inhibition can suppress inflammation and promote recovery when given 18 - 24 hours after brain ischemia [25,26], but the efficacy of this approach after TBI is not known. In the present study we used a controlled cortical impact model of TBI in the rat to determine whether delayed treatment with a PARP inhibitor, INO-1001, would suppress microglial and astrocyte activation and improve long-term recovery. INO-1001 was chosen for this study because it is an extremely potent PARP inhibitor and it has been approved for clinical trials [27].

## Materials and methods

The studies were approved by the San Francisco Veterans Affairs Medical Center Animal Care and Use Committee. Reagents were obtained from Sigma-Aldrich except where noted. Male Sprague-Dawley rats (250-300 g; Simonsen Laboratories), were housed two per cage on a 12 hour light/dark cycle with free access to food and water.

### Traumatic Brain Injury and drug treatment

A controlled cortical impact device (Pinpoint Precision Cortical impactor, Hatteras Instruments, Cary, NC) was used to produce a unilateral traumatic brain injury (TBI). Rats were anesthetized with intraperitoneal injections of ketamine (80 mg/kg) plus xylazine (8 mg/kg) and maintained at 37°C ± 0.5°C with a thermal mat throughout the surgical procedure. The anesthetized rats were placed in a stereotaxic frame with heads positioned to target the impact 3.5 mm left of bregma. A midline scalp incision was made and a circular craniotomy was made while maintaining integrity of the dura. Initial studies used a 2.5 mm diameter impactor programmed to 1.5 m/s velocity, 2.5 mm penetration depth, and 120 ms dwell time. In a later study, used for long-term behavioral endpoints, a 5 mm diameter impactor was used and the penetration depth was increased to 5 mm. Following the cortical impact, the skin overlying the site of injury was sutured closed and the animals were maintained at 37°C for 30 minutes and observed until recovered from anesthesia. Mortality rate was less than 5%. Sham-operated controls were subjected to the same surgical procedures except the cortical impact. The PARP inhibitor INO-1001 (Inotek Pharmaceuticals, Lexington, MA) was administered by intraperitoneal injection at a dose of 10 mg/kg in 1.0 - 1.5 ml sterile saline vehicle. Injections were begun 20 - 24 hours after TBI surgery, and every 24 hours thereafter except where otherwise noted.

### Immunohistochemistry

Rats were deeply anesthetized with isoflurane, given bilateral thoracotomy, and transcardially perfused with saline followed by 4% formaldehyde. Brains were post-fixed in 4% formaldehyde overnight at 4°C, cryoprotected in 20% sucrose for 2 days at 4°C, and rapidly frozen in dry ice. Serial 40 μm coronal sections were obtained using a cryostat. Four sets of 9 evenly spaced (640 μm apart) sections spanning the injured cortex were collected from each brain. Immunostaining was performed using mouse monoclonal antibody to CD11b (1:200, clone OX-42, Serotec, Oxford, UK), rabbit polyclonal antibody to glial fibrillary acidic protein (GFAP; 1:1000, Chemicon), and mouse monoclonal antibody to NeuN (1:1000, Chemicon). Incubations were made in blocking buffer containing 1% albumin, 2% normal serum and 0.3% Triton X-100. Antibody binding was imaged using fluorescent second antibodies and confocal microscopy (Zeiss LSM 510) with appropriated filter sets. For low magnification images, CD11b antibody binding was visualized by the DAB method [28], using biotin-conjugated second antibodies and the Vectastain Elite ABC reagent (Vector Laboratories). Controls prepared with primary antibody omitted showed no detectable fluorescence and minimal background DAB staining under the conditions employed.

### Histological outcome measures

The TBI lesion cavity was defined on each section as the area of brain devoid of NeuN staining (because both astrocytes and microglia, but not neurons, grow and move in to the lesion cavity). NeuN-stained sections were photographed, and the perimeter of each hemisphere was outlined using an image analysis program. The area of each lesioned hemisphere was then subtracted from the area of the contralateral hemisphere, and these values were summed and multiplied by the distance between sections to yield a lesion volume for each brain (modified from [29].

**Figure 1:**
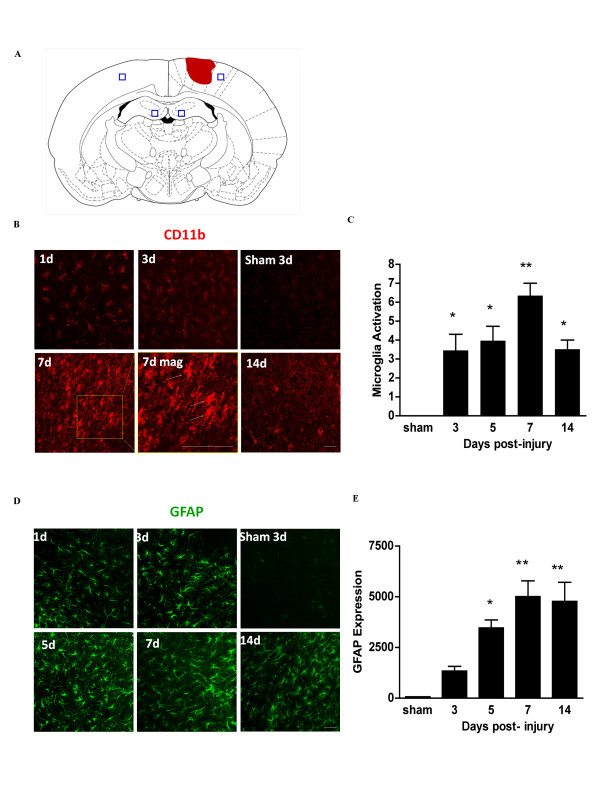
**Time course of microglia and astrocyte activation in cortex after controlled cortical impact**. (A) Diagram of coronal rat brain section showing relationship of lesion cavity (red) to regions photographed (black squares). (B) Confocal fluorescence images of CD11b immunoreactivity (red) in peri-lesion cortex at designated time points after TBI. Panel '7d mag' is a magnified view showing amoeboid morphology of the activated microglia (arrows). (C) Quantification of microglial activation. (D) Astrocytes are immunostained for GFAP (green), and staining is quantified in (E). Scale bars are 20 μm. n = 3 at each time point; * p < 0.05, ** p < 0.01 vs. sham.

Activation of microglia and astrocytes was evaluated in the lesioned and non-lesioned cortex and hippocampus on 4 coronal sections spaced 640 μm apart and centered on the lesion epicenter. Six 450 μm^2 ^areas were photographed for later analysis: an area centered 625 um lateral to the lesion edge and midway through the cortical depth; the homologous contralateral cortex; and the hippocampal dentate gyrus ipsilateral and contralateral to the cortical lesion (Figure 1A). The edge of the lesion was defined by complete lack of Neu N staining. The regions analyzed were offset from the edge by 400 μM to avoid introducing error by edge irregularities. Microglial activation was scored by evaluating the density of CD11b - positive microglia and microglial morphology (modified from [25], as detailed in Table 1. Astrocyte activation was quantified by measuring expression of glial fibrillary acidic protein (GFAP). GFAP expression level in each photographed area was calculated by multiplying the net area of GFAP staining by the intensity of GFAP staining, using the NIH ImageJ program.

**Table 1:** Scoring of microglial activation

Cell morphology (% amoeboid)	Score	Cell number (cells per 450 μm^2^)	Score
none	**0**	0-25	**0**

1-10%	**1**	25-50	**1**

10-25%	**2**	50-100	**2**

25-50%	**3**	100-150	**3**

> 50%	**4**	> 150	**4**

Microglia were identified by CD11b immunoreactivity. Scoring with the two criteria were combined to yield an aggregate microglia activation score (0-8).

### Behavioral outcome measures

The behavioral studies were performed over a 2-week interval beginning 8 weeks after TBI. Animals were housed in a dedicated behavioral study facility during this interval to minimize environmental changes associated with transfer between home cage and the testing arenas. All tests were performed and analyzed by individuals blinded to the experimental conditions. For the cylinder test, rats were placed in a clear plexiglass cylinder as described previously [30]. Normal rats touch the walls equally often with each forelimb, but hemi-paretic rats touch the walls less often with the affected limb. Rats were kept in the cylinder for 10 minutes and videotaped for scoring at a later date. The number of wall touches with each forelimb was scored for each rat. For the sticky tape test, a 0.5-inch diameter round adhesive tape was placed on the heel of each forepaw. The time to remove the tape from each paw was measured. Five trials were performed and the median value was calculated for each rat. Individual trials were timed out at 10 minutes. For the vermicelli test, rats were food-restricted for 48 hours and then given 5 pieces of dry vermicelli, each 7-cm in length, in their home cages as described previously [31]. Manipulation of the vermicelli was videotaped for later analysis. Normal, typical handling behavior involves holding the noodle asymmetrically with 2 paws, one as the guide paw and the other as the grasp paw, with the paws apart until the noodle is less than 3.5 cm in length. The behaviors scored on the videotapes were as follows: 1) Paws apart (a normal behavior) - paws placed assymetrically and apart with each adjustment; 2) Uses mouth (abnormal) - mouth is used to pull the noodle through the forepaws; 3) Single paw use (abnormal) - only one paw is used to manipulate the pasta; 3) Drop (abnormal) - the noodle is dropped shortly after eating begins; 4) Head tilt (abnormal) - head is tilted or face lowered while eating the noodle. The "hands apart" scoring was normalized to the number of total adjustments. The other measures were scored with a 1 if exhibited and a 0 if not for each of the five noodles consumed, and summed for each specified behavior. Rats were also scored for the total number of pauses counted while eating, the total number of paw adjustments made while eating, and the total time taken to consume the 5 noodles.

### Data analysis

Outcome measures were evaluated by observers blinded to experimental conditions. Results are presented as mean ± standard error. Statistical analyses were done by ANOVA followed by the Bonferroni test where comparisons were made between multiple groups or Dunnet's test where multiple groups were compared against a common control group. Composite scores for the vermicelli test data were obtained after first normalizing the score for each animal in each individual test to the mean score of the TBI + vehicle treatment group of that test.

## Results

### Time course of brain inflammation after TBI

**Figure 2:**
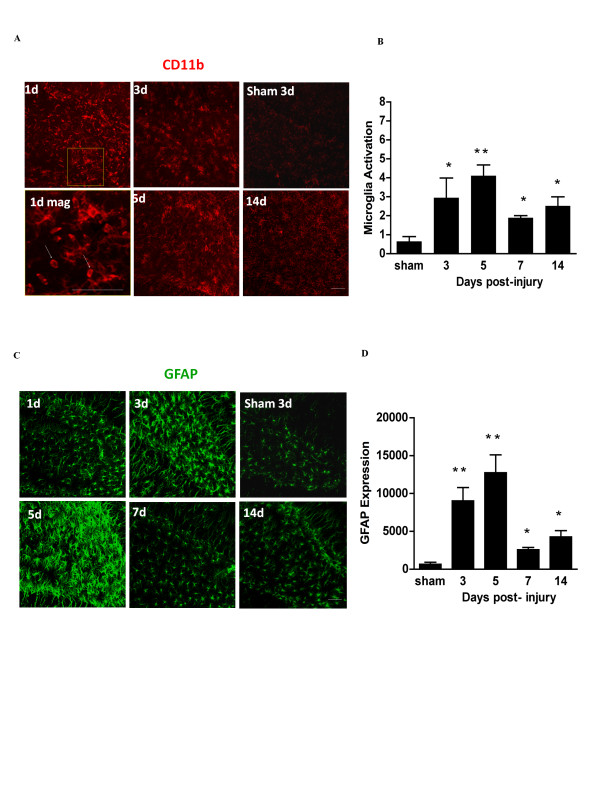
**Time course of microglia and astrocyte activation in the hippocampus after controlled cortical impact**. (A) Confocal immunofluorescence staining for activated microglia (CD11b) in the ipsilateral hippocampal dentate gyrus at designated time points after TBI. Panel '1d mag' is a magnified view showing amoeboid morphology of activated microglia (arrows). (B) Quantification of microglial activation. (C) Astrocytes are immunostained for GFAP (green), and staining is quantified in (D). Scale bars are 20 μm. n = 3 at each time point; * p < 0.05, ** p < 0.01 vs. sham.

The controlled cortical impact produced a necrotic lesion in the dorsal cortex that extended from the cortical surface to the deeper cortical layers, but not to the underlying corpus callosum (Figure 1A). We first evaluated the time course of microglia and astrocyte activation in the peri-lesion cortex and ipsilateral hippocampus after this injury to identify the period of peak inflammation. Activated microglia increase expression of the integrin CD11b and acquire an "amoeboid" morphology, with retracted, thickened processes and enlarged soma [1]. Activated astrocytes (also termed "reactive astrocytes") increase expression of GFAP and, like activated microglia, also exhibit retraction of processes and enlarged soma [32]. In peri-lesion cortex, activation of microglia and astrocytes peaked at about day 7 (Figure 1). There was no detectable activation in the contralateral cortex or in ipsilateral cortex remote from the impact site (not shown). In the ipsilateral hippocampus (dentate gyrus), microglial and astrocyte activation peaked by 5 days after TBI (Figure 2). Similar but less extensive activation was observed in the contralateral hippocampus (not shown).

### Post-treatment with INO-1001suppresses microglial activation after TBI

**Figure 3:**
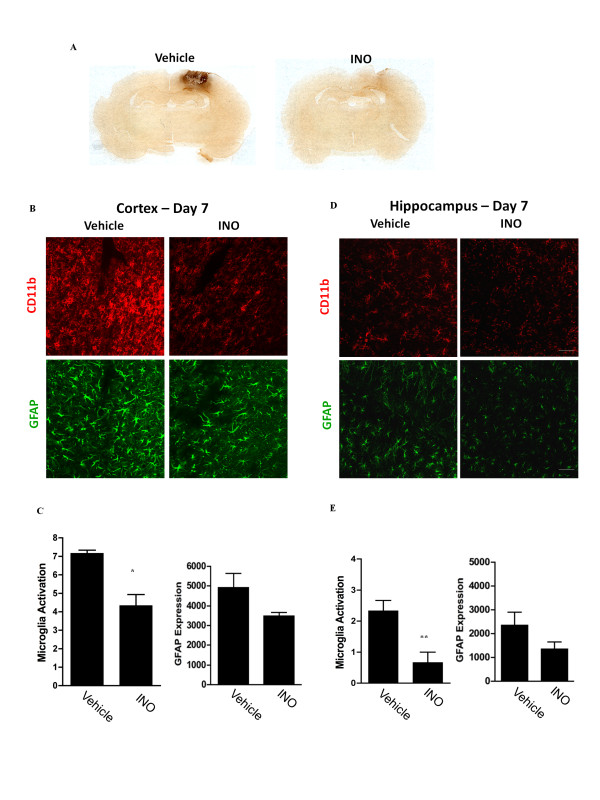
**Post-injury treatment with INO-1001 reduces microglia and astrocyte activation**. (A) Coronal rat brain sections showing microglial activation (CD11b immunostaining, brown) around the lesion sits. Rats were treated with vehicle or INO-1001 beginning 1 day after TBI, and brains were harvested at day 7. (B) Higher power confocal immunofluorescence staining of microglia (CD11b, red) and astrocytes (GFAP, green) in the peri-lesion cortex of rats treated with INO-1001 or vehicle. (C) Quantification of immunofluorescence. (D) Immunostaining in the ipsilateral hippocampal dentate gyrus of rats treated with INO-1001 or vehicle at day 7 post-TBI. (E) Quantifications of immunostaining in ipsilateral hippocampus. Scale bars are 20 μm. n = 3; *P < 0.05, **P < 0.01.

Separate groups of rats were then treated with INO-1001 (10 mg/kg) or vehicle for 7 days (encompassing the time of peak inflammation) to confirm that PARP inhibition can suppress TBI-induced brain inflammation. The drug was administered daily, with the first dose given 20 - 24 hours after injury. As expected, treatment initiated at this delayed time point did not affect the size of the necrotic lesion (not shown). However, rats receiving INO-1001 did exhibit reduced microglia activation in the peri-lesion cortex (Figure 3A,B,C). There was also a significant reduction in microglial activation in the ipsilateral hippocampus (Figure 3D,E). Reductions in astrocyte GFAP expression did not achieve statistical significance.

### No rebound inflammation after stopping treatment with PARP inhibitor

**Figure 4:**
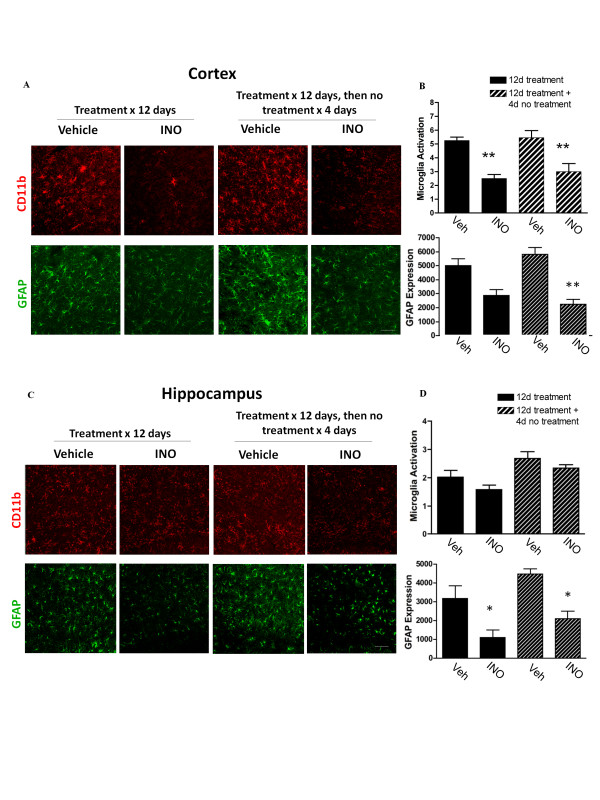
**No rebound inflammation after INO-1001 is discontinued**. Rats were treated with INO-1001 (or vehicle) beginning 1 day after TBI in one of two regimens: i) for 12 days, then euthanized, or ii) for 12 days followed by 4 days without treatment. (A) Confocal immunostaining of microglia (CD11b) and astrocytes (GFAP) in peri-lesion cortex. (B) Quantification shows no significant increase in microglial or astrocyte activation after stopping INO-1001. (C) Confocal immunostaining of microglia and astrocytes in the ipsilateral hippocampus dentate gyrus. (D) Quantification shows no increase in microglial or astrocyte activation after stopping INO-1001. Bars represents 20 μm. n = 4-5; *P < 0.05, **P < 0.01 vs. vehicle treatment.

We next considered the possibility that INO-1001 might induce a "rebound" inflammation when discontinued after the peak interval of microglial activation. One group of rats was treated with INO-1001 (or vehicle) for 12 days following TBI, at which time microglial activation had largely waned. A second group of rats was treated with INO-1001 (or vehicle) for 12 days, followed by 4 days of no treatment before brain harvest. The four-day interval was chosen because this interval is long enough to observe rebound inflammation when it occurs in other settings [33-36]. INO-1001 was in all cases initiated 20-24 hours after TBI. Results of these studies showed no rebound increase in microglial or astrocyte activation, in either cortex or hippocampus, following cessation of INO-1001 treatment (Figure 4).

### Improved neuronal survival in the peri-lesional cortex

**Figure 5:**
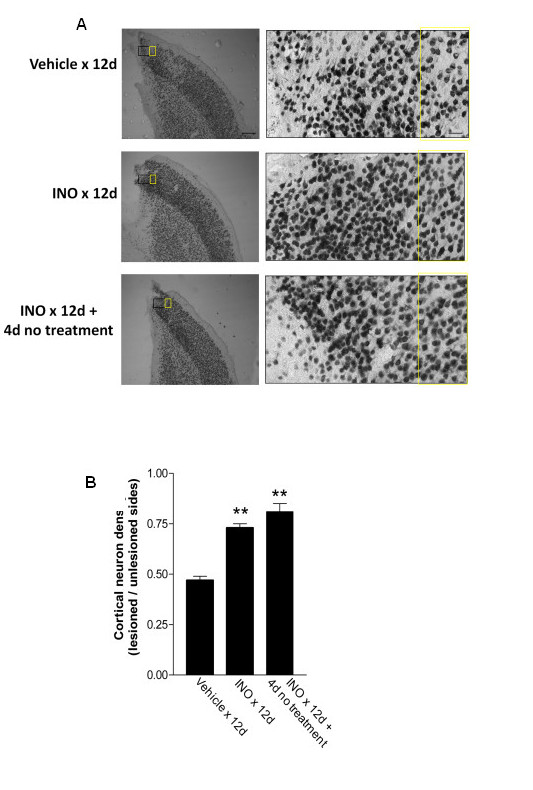
**Neuronal density in the peri-lesion area**. (A) Immunostaining for neurons (NeuN) in brain sections from rats treated as described in Fig. 4. The black and yellow rectangles in the low power views (left) show the regions magnified in the high power views (right). Cell counts were made in the regions identified in the yellow rectangle, 400 μm lateral to the lesion cavity edge, and in the homologous cortex of the contralateral hemisphere (not shown). Scale bars are 400 μm (low power views) and 40 μm (high power views). (B) Neuronal density was expressed in each animal as the ratio of densities in the lesioned and non-lesioned hemispheres. n = 4-5; *P < 0.05 vs. vehicle.

Microglial activation can lead to neuronal death [1,4,5]. We therefore evaluated neuronal survival in the peri-lesion cortex in the same brains evaluated for rebound inflammation. Neuronal density was found to be decreased in peri-lesion cortex relative to density in the contralateral (non-injured) cortex, and this decrease was attenuated in rats treated with INO-1001 (Figure 5).

### Effects of INO-1001 on long-term behavioral outcome measures

**Figure 6:**
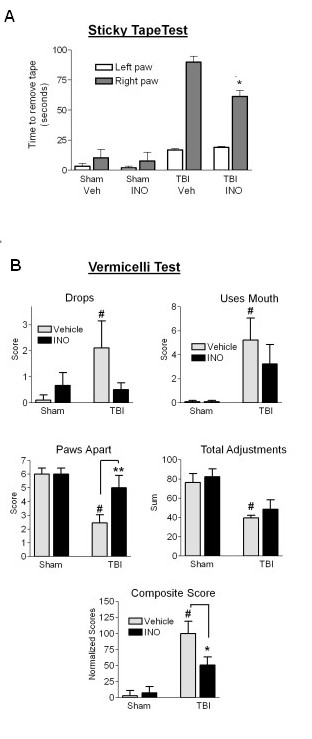
**Effect of INO-1001 treatment on forelimb dexterity**. Rats were subjected to TBI or sham surgery, and were treated with INO-1001 or vehicle for 12 days beginning 1 day after surgery. Behavioral assessments were made 8 weeks after TBI. (A) Time to remove an adhesive tape from the contralateal forelimb was markedly increased by TBI, and this time was reduced in the animals treated with INO-1001. n = 6-9; *P < 0.05. (B) Scoring of vermicelli test results. n = 6-9. *P < 0.05, P **P < 0.01, ^#^P < 0.05 vs. sham, vehicle-treated.

Studies using the initial TBI protocol showed no detectable forelimb dysfunction at the 8 week post-injury time point (data not shown). Consequently, a second study was performed using increased impactor depth and dwell time. Rats were subjected to either TBI or sham TBI, and treated with either vehicle or INO-1001 on post-surgery days 1 through 13 (12 days of treatment). Forelimb dexterity was evaluated 8 weeks after TBI using the cylinder test, the sticky tape test, and the vermicelli test. The cylinder test again showed no effect of TBI on paw preference (not shown). However, the sticky tape test showed a large increase in time taken to remove tape applied to the right forepaw in the TBI rats, and this increase was significantly attenuated in rats treated with INO-100 (Figure 6A*)*. Similarly, the vermicelli test, which is sensitive to deficits in fine coordination of the forepaws, showed abnormalities in several parameters in the TBI rats, and these were also attenuated by INO-1001 treatment (Figure 6B). Other parameters of the vermicelli test evaluated (head tilt, single paw use, and total feeding time, and total number of pauses) showed no significant difference between TBI and sham surgery groups and were therefore not included in the composite score analysis. Evaluation of the brains after behavioral testing (11 weeks after TBI) showed lesion cavity size to be not significantly different in the vehicle and INO-treated groups; 39.7 ± 3.1 and 43.7 ± 1.8 mm^3^, respectively.

## Discussion

The time course studies presented here showed that activation of both microglia and astrocytes peaked between 5 and 7 days after TBI in the peri-lesion cortex, and remained evident beyond 2 weeks. These observations are similar to those of prior reports [37,38]. This inflammatory response was suppressed by the PARP inhibitor, INO-1001. The reduced inflammation was accompanied by improved neuronal survival near the impact site and by improved performance on measures of forelimb dexterity carried out 8 weeks after injury. Importantly, treatment with the PARP inhibitor was not begun until 20 - 24 hours after TBI, a time window relevant to clinical treatment settings.

Neuronal death has been observed as a delayed effect of TBI [39,40]. Here, the neuronal loss co-localized with microglial activation, and both microglia activation and the neuronal loss were suppressed by INO-1001. This result, along with the known cytotoxicity of acutely activate microglia, suggests that the INO-1001 preserved neuronal survival by suppressing microglial activation; however, other potential mechanisms cannot be excluded.

One such potential mechanism is direct neuroprotection by INO-1001. Independent of its anti-inflammatory actions, INO-1001 and other PARP inhibitors can interrupt a programmed cell death pathway that is triggered by DNA damage [41-43]. Accordingly, prior studies have shown salutary effects of PARP inhibitors given shortly (within 60 minutes) after TBI [16,44-47]. A key and novel aspect of the present study is that PARP inhibition was not initiated until 20 - 24 hours after TBI. There were two reasons for this treatment delay. First, we were striving to identify a treatment approach that can be effective when initiated at time points relevant to clinical settings, which is generally several hours after injury. Second, we aimed to specifically test the idea that suppressing inflammation would improve outcome. A cyoprotective effect of acute PARP inhibition would confound testing of this idea because a reduction in initial injury severity would reduce injury-induced inflammation, independent of a direct anti-inflammatory effect. We cannot be absolutely certain that INO-1001 had no cytoprotective effects when administered 20 hours after injury, but this is rendered unlikely by the lack of an effect on necrotic lesion size in INO-1001 - treated groups, and by the lack of literature precedent for a cytoprotective effect of PARP inhibitors beyond 3 hours after injury.

In addition to neurotoxic effects, microglial activation can also have beneficial effects on outcome. The effects of microglial activation depend on the nature of the inciting stimulus, microglial heterogeneity, and other factors [3,8,48]. Temporal aspects of microglial activation may be particularly important, because initial microglia activation appears to be suited for stopping infection, while later activation and infiltration of blood-derived macrophages promotes repair and regeneration [8,49]. Thus, short periods of immunosuppression could, in principle, prevent the cytotoxicity associated with acute microglial activation without impairing the long-term beneficial effects of microglia activation on neurite outgrowth, remodeling, and functional recovery. This temporal consideration was a rationale for the 12 day treatment period employed here. This treatment period was based on the observation that the acute inflammatory response begins to wane after day 7 in this TBI model. We also considered the possibility that there may be a "rebound" in inflammation at the time of drug treatment ended, and that this rebound could negate any beneficial effects of limited immunosuppression. However, we found no evidence of a rebound effect on either astrocytes or microglial activation.

A technical limitation of this study is that there is no direct measure of PARP activity in the injured brains. PARP activity can be measured acutely after TBI by the accumulation of poly(ADP-ribose) polymer, the enzymatic produced of PARP-1 and other PARP species [15-17]. However, the polymer is quickly degraded by poly(ADP-ribose) glycohydrolase (PARG), such that polymer accumulation occurs only during the relatively transient post-injury interval when increased PARP activity has not yet been matched by increased PARG activity [9,13,14,26,43]. The inference here that the anti-inflammatory effects of INO-1001 result from a reduction in PARP activity that is otherwise be elevated for several days after TBI is based on the prior observations that TBI induces PARP activation [15-17], the high potency and selectivity of INO-1001 as a PARP inhibitor [16,27,45], and the ability of PARP inhibitors to block NF-kB - mediated inflammatory responses [12-14,20,25,26]. Nevertheless, the results presented cannot exclude the possibility that the anti-inflammatory effects of INO-1001 result from some other, unrecognized action of this drug.

Astrocyte activation is an important feature of glial scar formation during acute brain inflammation, but chronic astrogliosis may be deleterious to axonal regeneration after CNS injury [7]. The stimuli triggering astrocyte activation in TBI have not been identified, and it may be that astrocyte activation is driven at least in part by factors released from microglia. In the present studies, INO-1001 was found to significantly reduce astrocyte activation in some, but not all, of the experimental conditions examined. The reasons for this variability is not clear, but it suggests that astrocyte activation is not entirely dependent upon microglial activation and that astrocyte activation is regulated at least in part by PARP-1 - independent mechanisms.

Importantly, the histological effects of INO-1001 administration were accompanied by improvements in tests of forelimb dexterity. These tests were done 8 weeks after TBI, indicating a long-lasting drug effect. We speculate that only tests of fine forelimb dexterity are capable of identifying functional deficits at this late time point, given that the cylinder test, which evaluates proximal limb function, showed no abnormalities. The improved forelimb dexterity may be attributable to improved neuronal survival in the peri-lesion area, but other explanations are also possible. In particular, these studies did not evaluate effects of the PARP inhibitor of diffuse axonal injury or axonal survival after TBI.

## Conclusions

These findings support the idea that suppressing the innate brain inflammatory response during the first few days after TBI can improve histological and functional outcomes. Effective suppression of the inflammatory response can be achieved with a PARP inhibitor, and with a delay in treatment onset of 20-24 hours after TBI.

## List of abbreviations

GFAP: glial fibrillary acidic protein; ICAM-1: inter-cellular adhesion molecule 1; iNOS: inducible nitric oxide synthase; PARP: poly(ADP-ribose) polymerase; TBI: traumatic brain injury; TNFα: tumor necrosis factor alpha

## Competing interests

The authors declare that they have no competing interests.

## Authors' contributions

Joana C. d'Avila Ph.D. - designed studies, performed histological assessments, data analysis, and drafted the manuscript

Tina I Lam Ph.D - performed histological assessments and data analysis

Deborah Bingham, Ph.D. - performed and analyzed the behavioral studies

Jian Shi, Ph.D. - performed animal surgeries

Seok Joon Won Ph.D. - performed animal surgeries

Tiina M. Kauppinen Ph.D. - performed qualitative assessments of microglial activation

Stephen Massa M.D., Ph.D. - supervised animal surgeries and assisted in manuscript preparation Jialing Liu, Ph.D - supervised behavioral studies and analyses

Raymond A. Swanson MD - designed studies, supervised overall project, and performed final manuscript preparation
